# Mechanisms underlying the therapeutic effects of 4-octyl itaconate in treating sepsis based on network pharmacology and molecular docking

**DOI:** 10.3389/fgene.2022.1056405

**Published:** 2022-11-03

**Authors:** Maolin Chen, Wenxing Su, Fangling Chen, Tianlun Lai, Yilun Liu, Daojiang Yu

**Affiliations:** ^1^ Department of Burn and Plastic Surgery, The First Affiliated Hospital of Chengdu Medical College, Chengdu, China; ^2^ Department of Plastic and Burn Surgery, The Second Affiliated Hospital of Chengdu Medical College (China National Nuclear Corporation 416 Hospital), Chengdu, China; ^3^ School of Clinical Medicine, Chengdu Medical College, Chengdu, China; ^4^ Department of Anesthesiology, West China Hospital of Sichuan University, Chengdu, China

**Keywords:** network pharmacology, molecular docking, sepsis, hub gene, 4-octyl itaconate

## Abstract

**Objective:** Through network pharmacology and molecular docking technology, the hub genes, biological functions, and signaling pathways of 4-Octyl itaconate (4-OI) against sepsis were revealed.

**Methods:** Pathological targets of sepsis were screened using GeneCards and GEO databases. Similarly, the pharmacological targets of 4-OI were obtained through Swiss TargetPrediction (STP), Similarity ensemble approach (SEA), and TargetNet databases. Then, all the potential targets of 4-OI anti-sepsis were screened by the online platform Draw Venn diagram, and the hub genes were screened by Cytoscape software. The identified hub genes were analyzed by GO and KEGG enrichment analysis, protein interaction (PPI) network, and molecular and docking technology to verify the reliability of hub gene prediction, further confirming the target and mechanism of 4-OI in the treatment of sepsis.

**Results:** After the target screening of 4-OI and sepsis, 264 pharmacological targets, 1953 pathological targets, and 72 genes related to 4-OI anti-sepsis were obtained, and eight hub genes were screened, namely MMP9, MMP2, SIRT1, PPARA, PTPRC, NOS3, TLR2, and HSP90AA1. The enrichment analysis results indicated that 4-OI might be involved in regulating inflammatory imbalance, immunosuppression, and oxidative stress in developing sepsis. 4-OI protects multiple organ dysfunction in sepsis by acting on hub genes, and MMP9 is a reliable gene for the prognosis and diagnosis of sepsis. The molecular docking results showed that 4-OI binds well to the hub target of sepsis.

**Conclusion:** 4-OI plays an antiseptic role by regulating MMP9, MMP2, SIRT1, PPARA, PTPRC, NOS3, TLR2 and HSP90AA1. These Hub genes may provide new insights into follow-up research on the target of sepsis treatment.

## 1 Introduction

Sepsis is a systemic inflammatory response disease that leads to life-threatening organ dysfunction with the imbalance of the host response to infection (Kim et al., 2019). Although the research on sepsis’s treatment and pathophysiological mechanism has developed rapidly in recent years, it is still one of the diseases with the highest mortality worldwide (Mushtaq and Kazi, 2022). The Institute for Health Metrics and Evaluation (IHME) of the University of Washington conducted a statistical analysis of global, regional, and national sepsis incidence and mortality from 1990 to 2017. The results showed that as of 2017, about 48.9 million sepsis cases were recorded worldwide, of which 11 million died of sepsis, with a mortality rate as high as 22.5% (Rudd et al., 2020). A retrospective cohort study statistically analyzed 50.49 million adult patients hospitalized between 2010 and 2017, and the results show that although the length of hospital stay in patients with sepsis has improved, however, the incidence and mortality of sepsis in hospitalized patients are increasing every year (Imaeda et al., 2021). In recent years, although domestic and foreign scholars have carried out much research on the early identification and diagnosis, pathogenic mechanism, prognosis, and treatment of sepsis, as shown by the epidemiology of sepsis, the mortality rate of its hospitalized patients is still increasing every year. Therefore, finding more effective treatments for sepsis patients remains a serious challenge. In addition, factors such as the complexity of the pathogenesis of sepsis, population heterogeneity, and the lack of specific biomarkers have increased the difficulty for clinicians in diagnosing and treating sepsis and significantly increased the economic burden on the medical system and patients (Cheng et al., 2020). Therefore, there is an urgent need to find an effective method for treating sepsis.

The first description of itaconate dates back to the 19th century, but it was not until nearly the last decade that people had a more comprehensive and profound understanding of it (Cordes and Metallo, 2021). In recent years, itaconate has been a crucial immune metabolite in mammalian immune cells. Itaconate has recently been a crucial immune metabolite discovered in mammalian immune cells. When the body is invaded and stressed by pathogens, the cells will synthesize and secrete itaconate. It has been reported to be vital in immune regulation, antioxidant, antibacterial, and antiviral (Weiss et al., 2018; Hooftman and O'Neill, 2019). The current mechanistic research on treating sepsis with itaconate has achieved impressive results, including its effects on the Nrf2 pathway, the glycolytic pathway, and the NLRP3 inflammasome (Liao et al., 2019; Hooftman et al., 2020; Song et al., 2020). Lin et al. showed that 4-OI, a membrane-permeable itaconate derivative, may treat sepsis by modulating the complex interplay between metabolism, immunity, and inflammation (Lin et al., 2021). Furthermore, given that itaconate is produced during the natural immune response, its toxicity is likely to be very low, which may be as expected as the discovery of antibiotics in the first place (O'Neill and Artyomov, 2019). Itaconate and its derivatives have been found to have great potential in treating inflammatory diseases such as sepsis, psoriasis, gout, and rheumatoid arthritis (Daly et al., 2020; Peace and O'Neill, 2022). In addition, itaconic-CoA, a metabolite of itaconate, can kill conjugating mycobacteria by inhibiting B12-dependent methylmalonyl-CoA mutase (MUT) activity (Ruetz et al., 2019).

Itaconate may become an effective drug for the clinical treatment of sepsis shortly. However, the current research on the mechanism of itaconate anti-sepsis mainly focuses on animal and cell experimental models, and its molecular mechanism has not been fully elucidated. Therefore, more research is needed to elucidate the anti-sepsis targets and mechanisms of itaconate so that itaconate can be put into clinical trials as soon as possible to treat sepsis patients. 4-OI is a cell-permeable itaconate derivative with similar thiol reactivity to itaconate, which can react with or without LPS stimulation. The increased level of itaconate through hydrolysis has promoted 4-OI as a suitable surrogate for studying the biological function of itaconate (Mills et al., 2018; Swain et al., 2020). Combining chemical genomics and network biology, this new network pharmacology approach to observing drug action from the perspective of network biology can allow us to discover better drugs to treat complex diseases (Hopkins, 2007). Molecular docking technology is a crucial tool to help us better understand the interaction between compounds and molecular targets (Pinzi and Rastelli, 2019). Therefore, based on network pharmacology and molecular docking technology, this study revealed the hub genes and mechanisms of 4-OI against sepsis.

## 2 Materials and methods

### 2.1 Prediction of 4-octyl itaconate pharmacological targets

The SMILES (Simplified Molecular Linear Input Specification) is obtained from the PubChem (https://pubchem.ncbi.nlm.nih.gov/) database with the search term “4-Octyl itaconate”, which is an ASCII string that explicitly describes the three-dimensional chemistry of molecules structure), and then input the retrieved SMILES into Swiss TargetPrediction (http://www.swisstargetprediction.ch/), Similarity ensemble approach (https://sea.bkslab.org/), TargetNet (http://targetnet.scbdd.com/) database to obtain the pharmacological target of 4-OI, the probability is set to >0. The species chosen was “*Homo sapiens*” and the search results were filtered and deduplicated.

### 2.2 Prediction of pathological targets in sepsis

Enter “sepsis” in the GEO (http://www.ncbi.nlm.nih.gov/geo) database to search, select the species as “*Homo sapiens*” and download the microarray data set GSE69063 based on-chip sequencing (including 33 healthy samples, 57 samples of sepsis), and then used the online tool GEO2R to obtain differentially expressed genes between the healthy group and sepsis. The screening conditions were set as |logFC|>1, *p*-value<0.05. GeneCards (https://www.genecards.org/) screening: database to “sepsis” as the keyword search, set score >1 target for sepsis targets. It is a database that provides detailed information on all currently annotated and predictable genes in humans. The targets obtained from the Genecards and GEO database after screening and deduplication are potential pathological targets of sepsis.

### 2.3 Acquisition and analysis of 4-octyl itaconate antiseptic intersection target

Using the online platform, draw a Venn diagram (http://bioinformatics.psb.ugent.be/) to obtain the intersection targets of 4-OI in anti-sepsis, and then import the intersection targets into STRING (https://cn.string-db.org/) (Version 11.5) data analysis platform for PPI analysis, with species set to “*Homo sapiens*” and protein interaction score set to a high confidence level of 0.700. Then use the online analysis tool Kobas 3.0 (http://kobas.cbi.pku.edu.cn/) to perform KEGG and GO analysis on the intersection targets.

### 2.4 Screening and analysis of hub targets

The hub targets of 4-OI in treating sepsis were screened by Cytohubba, an analysis tool of Cytoscape (version 3.9.0) software. Of course, we use six topological analysis methods (MCC, MNC, Degree, Stress, EPC, Bottleneck) commonly used in Cytohubba analysis tools to evaluate and select hub targets. Then, the co-expression network and functional modules of hub genes are constructed through the GeneMANIA (http://www.genemania.org/) database, mainly used to generate hypotheses about gene functions and analyze gene lists. It is a reliable tool for getting in touch. The hub targets were then analyzed by KEGG and GO using the online analysis tool Kobas 3.0.

### 2.5 Hub gene verification

In order to further verify the reliability of the hub genes, we verified the accuracy of the obtained hub genes in other datasets, searched the GEO database with the keyword “sepsis” and finally screened the dataset GSE95233. The obtained dataset was divided into three groups, sepsis survivor-control group (SS-C), sepsis non-survivor-control group (SN-C), and sepsis survivor-sepsis Non-Survivor Group (SS-SN). We used R software to perform differential gene expression analysis of hub genes in SS-C, SN-C, and SS-SN groups.

### 2.6 Multi-organ expression verification of hub genes

Sepsis patients are often accompanied by multiple organ dysfunction. Therefore, we explored whether the essential genes were differentially expressed in sepsis-induced organ dysfunction, searched the GEO database with “sepsis” and “organ” as keywords, and finally obtained datasets GSE60088 and GSE5663. The obtained data sets were divided into four groups: sepsis-liver injury-control group (SLi-C), sepsis-lung injury-control group (SLI-C), sepsis-kidney injury-control group (SKi-C) and sepsis-spleen injury-control group (SSp-C). We used R software to analyze the differential gene expression of hub genes in SLi-C, SLu-C, SKi-C, and SSp-C groups, respectively.

### 2.7 Diagnostic ability of hub genes

The ROC curve analyzed the diagnostic ability of hub genes for sepsis. Using the dataset GSE95233 as a sample, we used R software to draw diagnostic ROC curves for eight hub genes.

### 2.8 Molecular docking verification-AutoDock vina

In order to accurately obtain the PDB ID of the hub target, we converted the hub gene ID through the UniProt (https://www.uniprot.org/) database. We then downloaded the 3D crystal structure of the hub protein using the PDB (https://www.rcsb.org/) database. It was chosen to be saved in PDB format as a protein receptor. At the same time, download the 2D structure of 4-OI from the PubChem database, save it in “SDF” format, and use OpenBabel (version 2.4.1) software to convert it to PDB format as a small molecule ligand. We use PyMOL software to remove water molecules and original ligands from protein molecules. Then, AutoDockTools (version 1.5.6) software was used to convert the PDB format files of proteins and small molecules into pdbqt format, including some operations: hydrogenation, charge calculation, atom type addition, and determination of torque center (root). Adjusting the X, Y, and Z centers on the original ligands of the different receptors. The central network box of MMP9, MMP2, SIRT1, PPARA, PTPRC, NOS3, TLR2, and HSP90AA1 were (9.86,11.269,4.001), (2.88,5.089,28.933), (2.88,5.089,28.933), respectively. (20.568, 3.416, 26.429), (11.386, 12.69, 15.397), (6.722, 0.649, 5.14), (9.86, 11.269, 4.001), (1.516, 14.704, 14.946) and (0.605, 30.053,20.297). Finally, the protein receptor and the small molecule ligand are docked by AutoDock vina (version 1.1.2). The size of the docking binding energy of the two indicates the strength of the binding activity. Small molecule ligands can bind spontaneously, and the smaller the binding energy, the better the binding activity and the stronger the binding stability. PyMol (version 2.2.0) software visualized the docking results with minimal receptor-ligand binding energy.

### 2.9 Molecular docking verification-discovery studio

In order to further improve the reliability of predicted core targets, we used the Discovery Studio 2019 Client software (protein structure analysis software, widely used in molecular docking) to remove water molecules, hydrogenation, apply forcefield, clean protein, and other operations on the core targets, and then obtain macromolecular receptors. Hydrogenation and applying forcefield is performed on the core target’s active ligand. The macromolecular receptor is molecularly docked with the treated active ligand. The RMSD value is obtained (RMSD represents the structural difference between two molecules (or between two states of the same molecule), a smaller value indicates a more accurate docking method). After the reliability verification of the docking software, we began to do molecular docking between the core target and the drug. Before molecular docking, we performed operations such as removing water molecules, deleting ligand groups, hydrogenation, cleaning proteins for the hub targets, and preparing ligands for small-molecule drugs.

### 2.10 Statistical analysis

All statistical analysis in this paper was completed with Sangerbox 3.0 platform (Shen et al., 2022), an online bioinformatics analysis tool developed based on the R language. Peers have widely recognized its accuracy and authenticity.

## 3 Result

### 3.1 Sepsis and 4-octyl itaconate target identification

**FIGURE 1:**
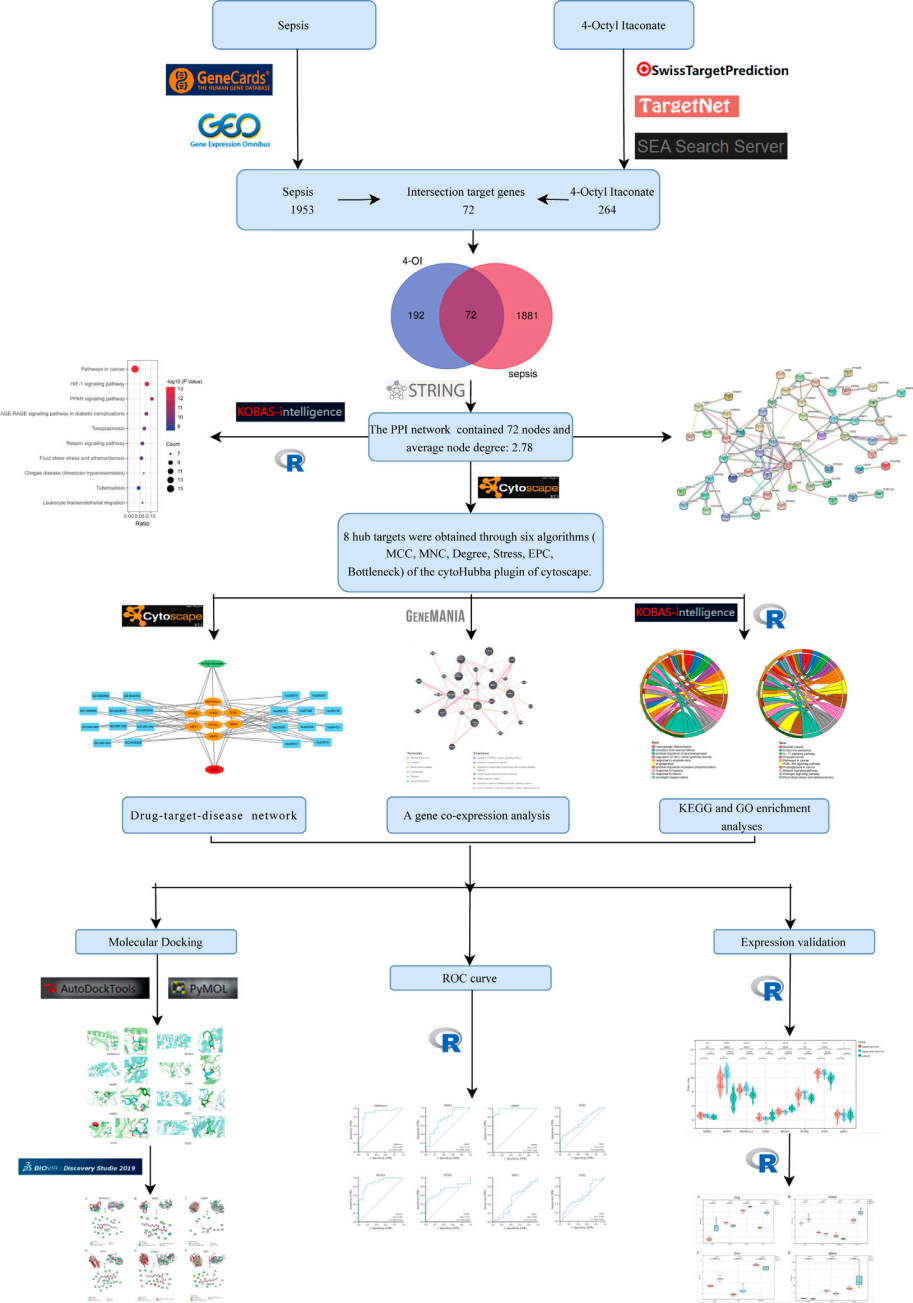
The design flow chart of this study.

**FIGURE 2:**
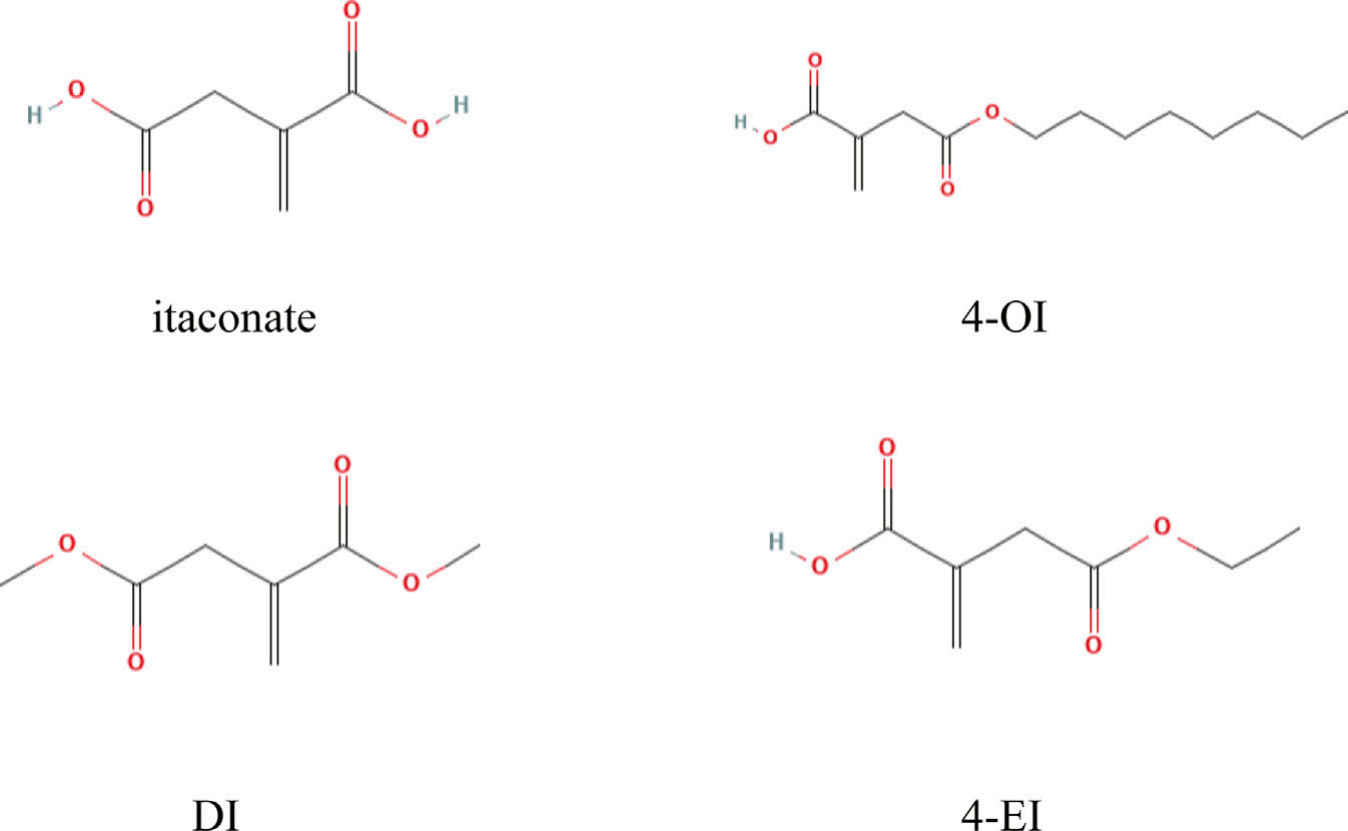
Chemical structures of itaconate and its derivatives.

The flow chart of this study design is shown in Figure 1. A limited selection of the obtained microarray dataset GSE69063 resulted in 1,193 differentially expressed genes (DEGs), regarded as pathological targets of sepsis (Supplementary Table S1), whether up-regulated or down-regulated. In addition, 844 sepsis-related pathological targets (Supplementary Table S2) were obtained through the GeneCards database. Finally, a total of 1963 potential targets were obtained after screening and deduplication of the two groups of sepsis targets. A total of 288 targets of 4-OI were obtained from STP, SEA, and TargetNet databases, and 264 targets were obtained after screening and deduplication (Supplementary Tables S3–S5). The chemical structures of itaconate and its derivatives are shown in Figure 2.

### 3.2 Analysis of functional characteristics of intersection targets of sepsis and 4-OI

**FIGURE 3:**
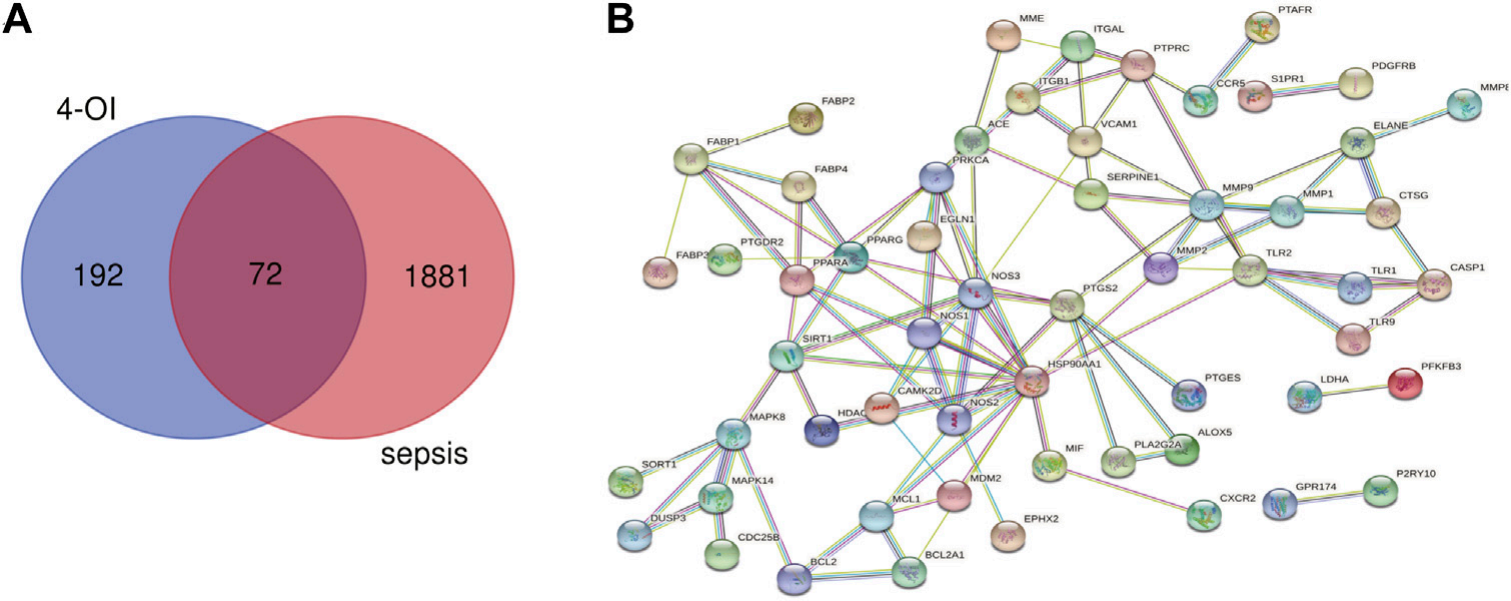
Venn diagram and PPI network were drawn for all 4-OI antiseptic targets. **(A)** The Venn diagram identified a total of 72 crossover targets. **(B)** The PPI cross-target network is constructed using Cytoscape software, and the interaction score is set to a high confidence level of 0.700.

**FIGURE 4:**
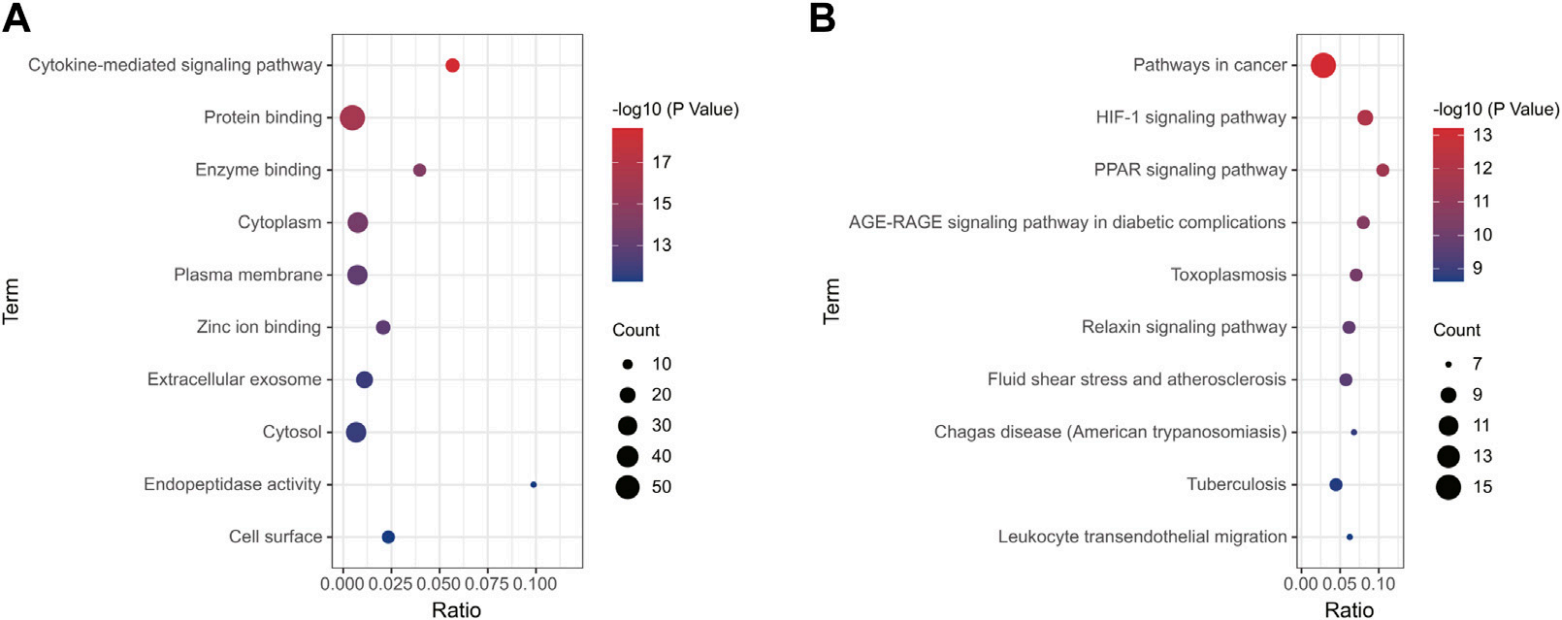
Enrichment analysis of all potential targets of 4-OI anti-sepsis. The GO and KEGG enrichment analysis of potential targets shows the enrichment data of the first 10 GO and KEGG. **(A,B)** The circle size represents the number of genes involved, and the abscissa represents the frequency of the genes involved in the term total genes.

Using the online tool Draw Venn diagram to draw a Venn diagram for the targets related to sepsis and 4-OI (Figure 3A), 72 potential targets were obtained. Targets with a protein interaction score >0.7 were imported into the STRING database to generate a PPI network (Figure 3B), which contained 72 nodes, average node degree: 2.78, and PPI enrichment *p*-value < 0.01. GO and KEGG pathway enrichment analysis was performed to determine the biological functions of the intersection targets. The results of GO analysis (Figure 4A) showed that these genes were mainly enriched in Endopeptidase activity, Cytoplasm, Exosomes, Zinc ion binding, Plasma membrane, Cytoplasm, Enzyme binding, Protein binding, Cytokine-mediated signaling, and Cell surface. KEGG analysis results include (Figure 4B): Pathways in cancer, Leukocyte transendothelial migration, Tuberculosis, Chagas disease (American trypanosomiasis), Fluid shear stress and Atherosclerosis, Relaxin signaling pathway, Toxoplasmosis, AGE-RAGE signaling pathway in diabetic complications, PPAR signaling pathway, HIF-1 signaling pathway.

### 3.3 Screening and analysis of hub targets

**FIGURE 5:**
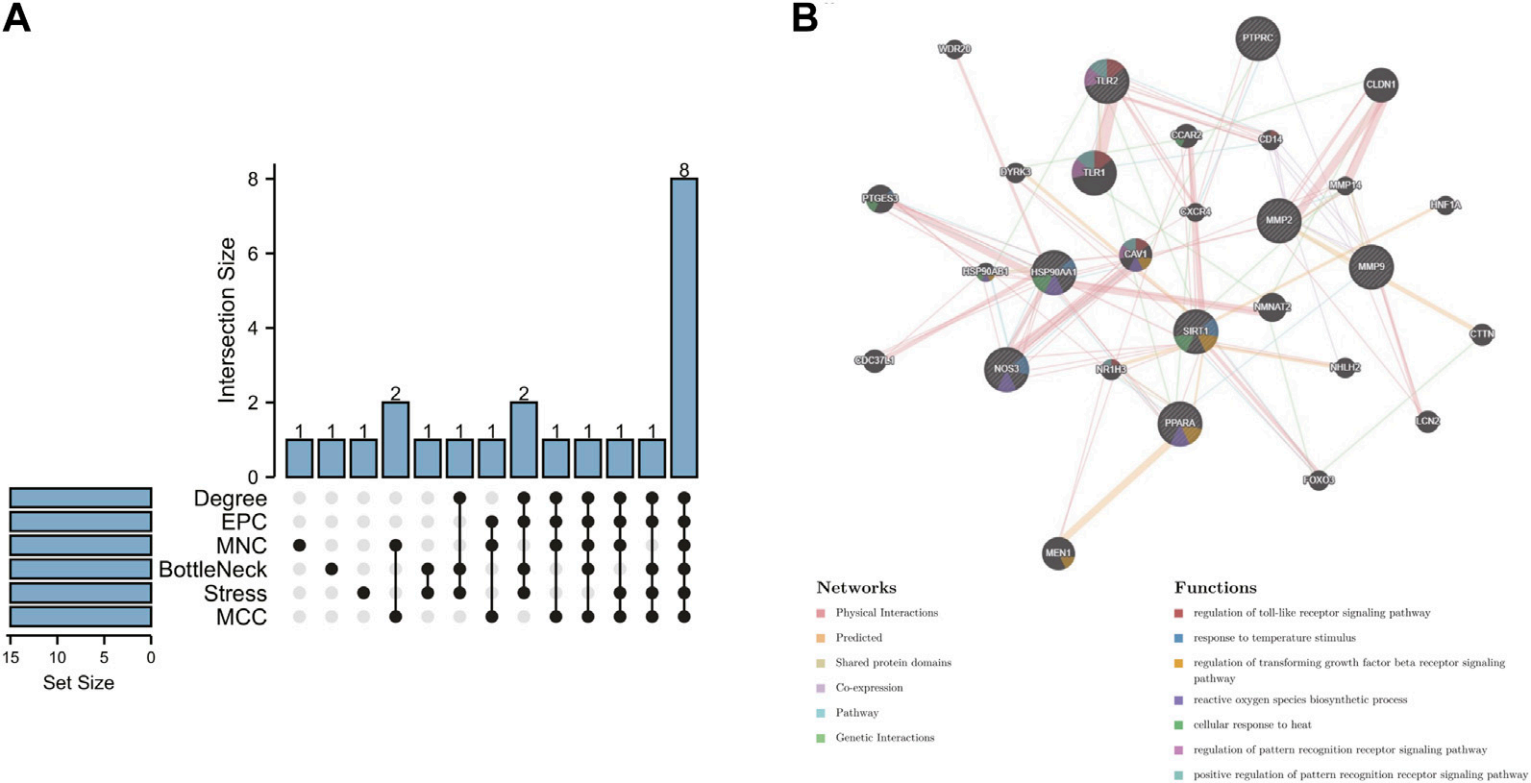
4-OI anti-sepsis Venn map and Hub gene coexpression network map. **(A)** The Venn map drawn by the R language package shows that six algorithms screened eight crossover Hub genes. **(B)** GeneMANIA analyzed the hub gene and its coexpression genes.

**FIGURE 6:**
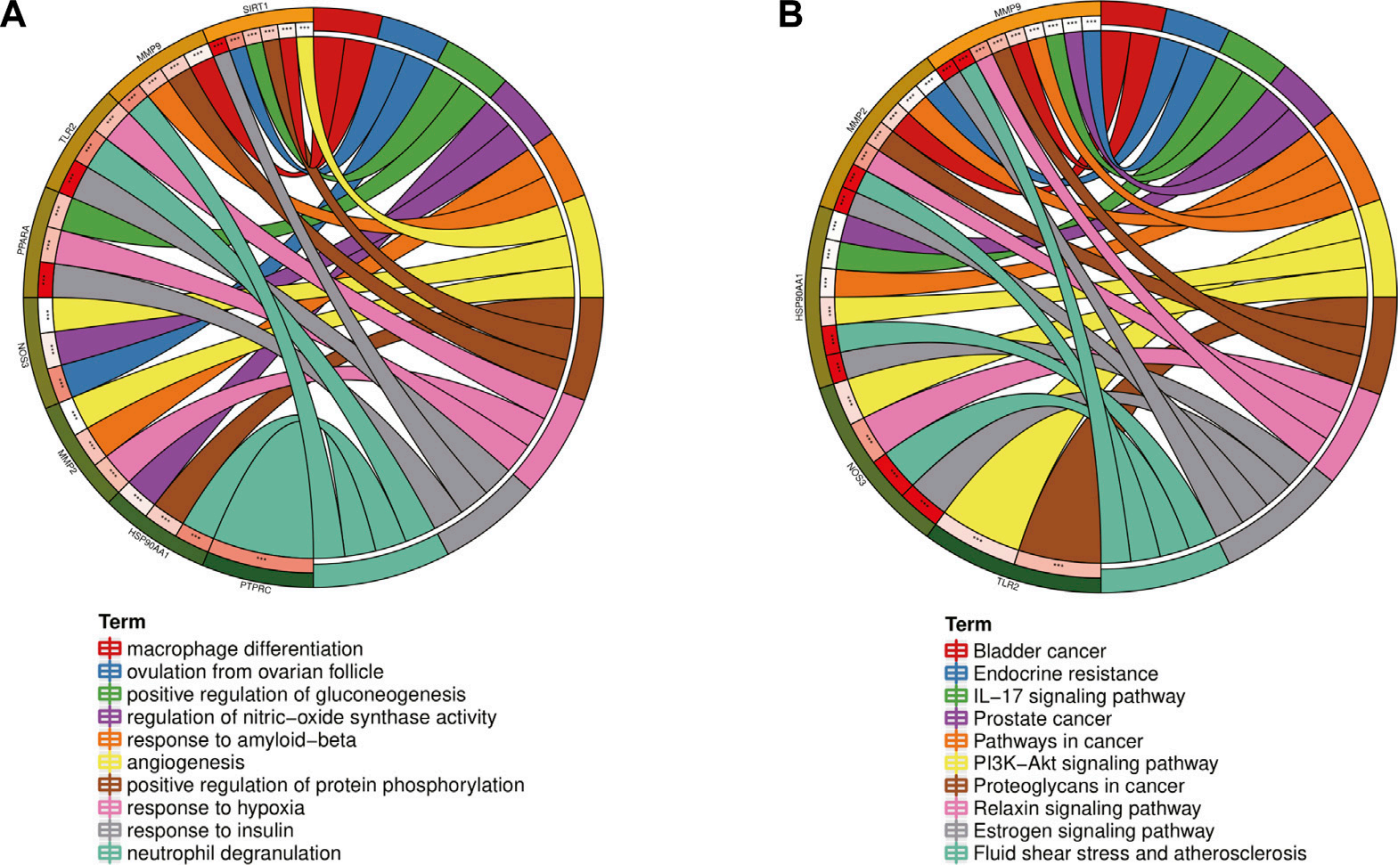
GO and KEGG enrichment analysis of the Hub gene showed the top 10 enrichment data. **(A,B)**The outermost part of the Circro circle shows the genes of the first ten enrichment items, and the inner circle on the left represents the significant *p*-value of the corresponding gene pathway.

**FIGURE 7:**
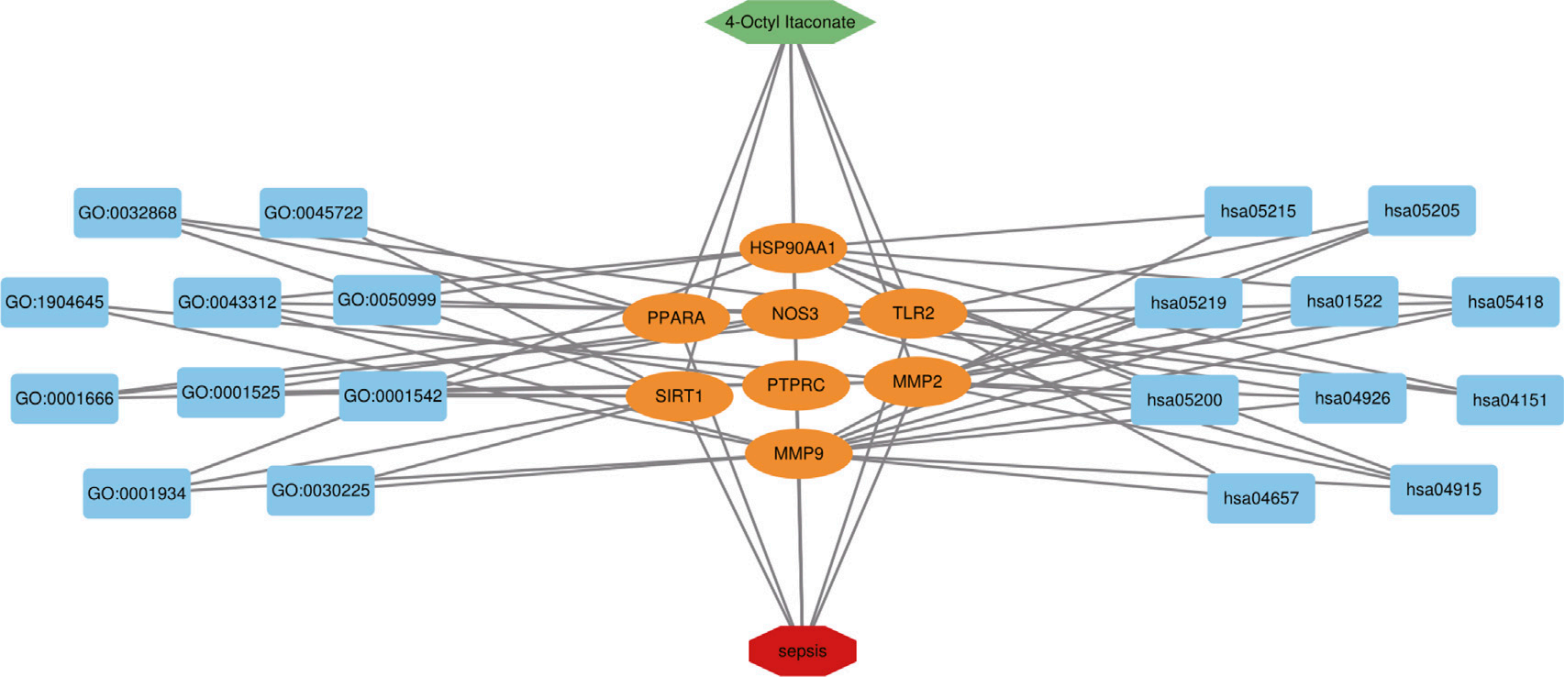
4-OI-target-sepsis detailed interaction network diagram. The dark blue rectangle on the left represents the first ten biological processes of the antiseptic effect of 4-OI. The dark blue rectangle on the right represents the first 10 KEGG signaling pathways of the 4-OI antiseptic effect. The orange oval in the middle represents the antiseptic target of 4-OI.

In order to further explore the hub genes of 4-OI against sepsis, we calculated the top 15 hub genes for each algorithm using six topological analysis methods in the Cytohubba analysis tool (Table 1 for details). Then, after taking the intersection, a total of eight hub genes were obtained, and their full names, functions, and Scores were displayed (Table 2), and a Venn diagram was drawn (Figure 5A), including MMP9, MMP2, SIRT1, PPARA, PTPRC, NOS3, TLR2, and HSP90AA1. Co-expression network and function analysis of hub genes based on the GeneMANIA database showed a complex PPI network (Figure 5B). The physical interactions of 51.21% predicted 17.33%, shared protein domains of 15.73%, co-expression of 9.91%, and pathway of 5.21%. The GO and KEGG enrichment analysis selects the top 10 results according to the significant difference in enrichment and visualizes these analysis results through Circro circles. The results of GO analysis related to hub gene include (Figure 6A): Macrophage differentiation, Follicular ovulation, Positive regulation of gluconeogenesis, Regulation of nitric oxide synthase activity, Response to amyloid-beta, Angiogenesis, Positive regulation of protein phosphorylation, Response to hypoxia, Response to insulin, Neutrophil degranulation. KEGG analysis results related to hub genes include (Figure 6B): Bladder cancer, Endocrine resistance, IL-17 signaling pathway, Prostate cancer, Cancer pathways, PI3K-Akt signaling pathway, Proteoglycans in cancer, Relaxin signaling, Estrogen signaling, Fluid shear stress, Atherosclerosis. Cytoscape software was used to construct the interaction network visualization map of 4-OI anti-sepsis hub target-related functions and pathways (Figure 7).

**TABLE 1:** The top 15 hub genes rank in CytoHubba.

MCC	MNC	Degree	BottleNeck	Stress	EPC
HSP90AA1	HSP90AA1	HSP90AA1	HSP90AA1	HSP90AA1	HSP90AA1
NOS3	MMP9	NOS3	MMP9	SIRT1	NOS3
MMP9	NOS3	MMP9	PTGS2	NOS3	NOS2
NOS1	NOS2	TLR2	TLR2	PPARG	PRKCA
NOS2	SIRT1	PPARG	SIRT1	TLR2	SIRT1
PRKCA	MMP2	PTGS2	MMP2	MMP9	PPARG
TLR2	PRKCA	NOS2	MAPK8	PTGS2	PPARA
SIRT1	MCL1	SIRT1	VCAM1	MAPK8	MMP9
PTPRC	NOS1	PTPRC	NOS3	PPARA	NOS1
VCAM1	MMP1	VCAM1	PTPRC	PTPRC	VCAM1
MMP2	PPARA	PRKCA	PRKCA	FABP1	TLR2
MMP1	CASP1	PPARA	PPARA	VCAM1	PTGS2
MCL1	CTSG	MMP2	PPARG	MMP2	MMP2
PPARA	TLR2	MCL1	FABP1	NOS2	MCL1
CTSG	PTPRC	FABP1	CCR5	ACE	PTPRC

**TABLE 2:** The details of the hub genes.

Gene symbol	Full name	Gene score	Function
TLR2	Toll Like Receptor 2	11.07	Cooperates with LY96 to mediate the innate immune response to bacterial lipoproteins and other microbial cell wall components
MMP9	Matrix Metallopeptidase 9	4.38	Matrix metalloproteinase that plays an essential role in local proteolysis of the extracellular matrix and in leukocyte migration
NOS3	Nitric Oxide Synthase 3	3.24	Produces nitric oxide (NO) which is implicated in vascular smooth muscle relaxation through a cGMP-mediated signal transduction pathway
PTPRC	Protein Tyrosine Phosphatase Receptor Type C	3.04	Protein tyrosine-protein phosphatase required for T-cell activation through the antigen receptor
SIRT1	Sirtuin 1	2.38	Involved in the coordination of multiple cellular functions such as cell cycle, response to DNA damage, metabolism, apoptosis and autophagy
HSP90AA1	Heat Shock 01	2.31	promotes the maturation, structural maintenance and proper regulation of specific target proteins involved for instance in cell cycle control and signal transduction
MMP2	Matrix Metallopeptidase 2	1.91	involved in diverse functions such as remodeling of the vasculature, angiogenesis, tissue repair, tumor invasion, inflammation, and atherosclerotic plaque rupture
PPARA	Peroxisome Proliferator Activated	1.90	SLigand-activated transcription factor., Key regulator of lipid metabolism

### 3.4 Core gene verification and diagnosis

**FIGURE 8:**
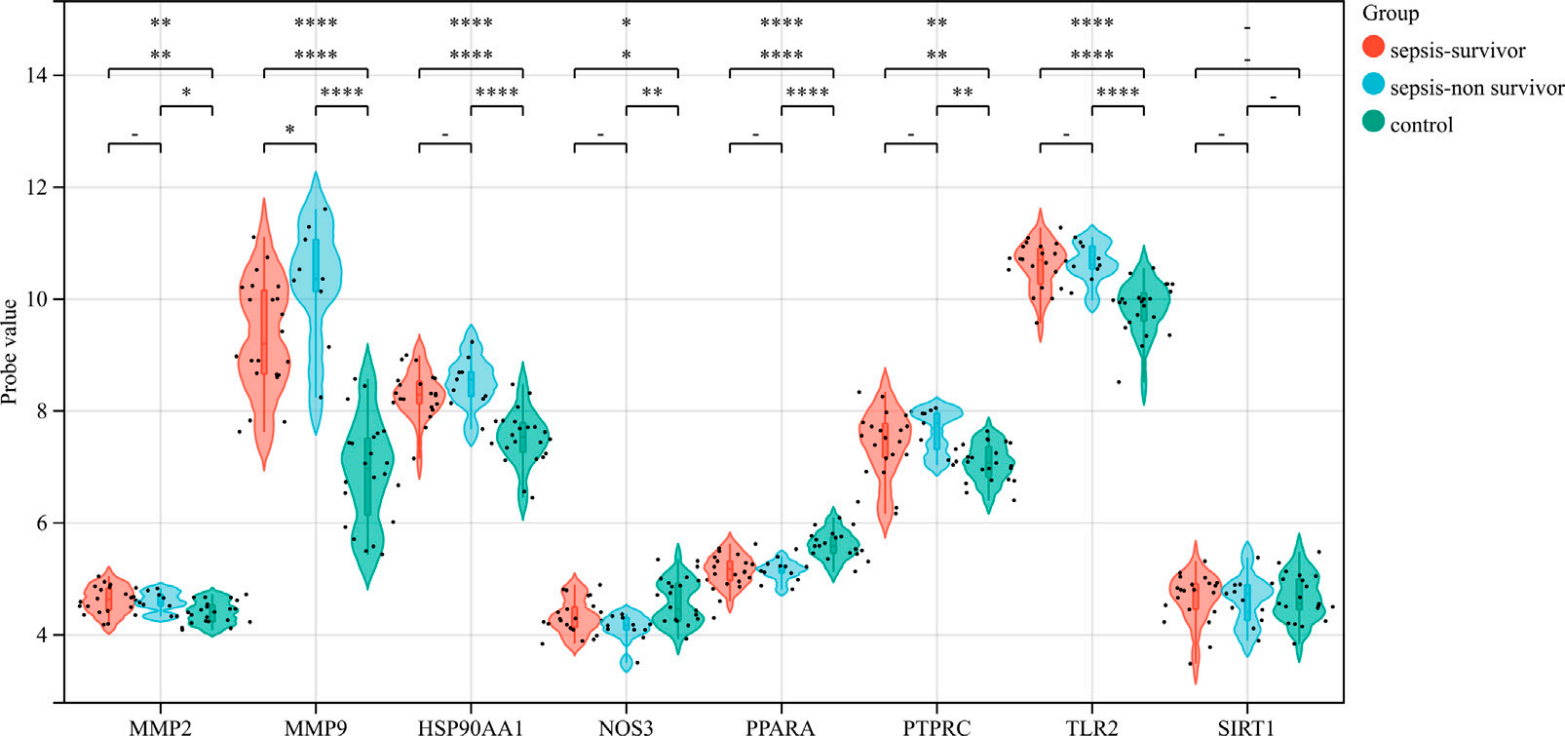
Eight hub genes were found in the sepsis survivor-control group (SS-C), sepsis non-survivor-control group (SN-C), and sepsis survivor-sepsis non-survivor group (SS- SN) differential expression analysis.

**FIGURE 9:**
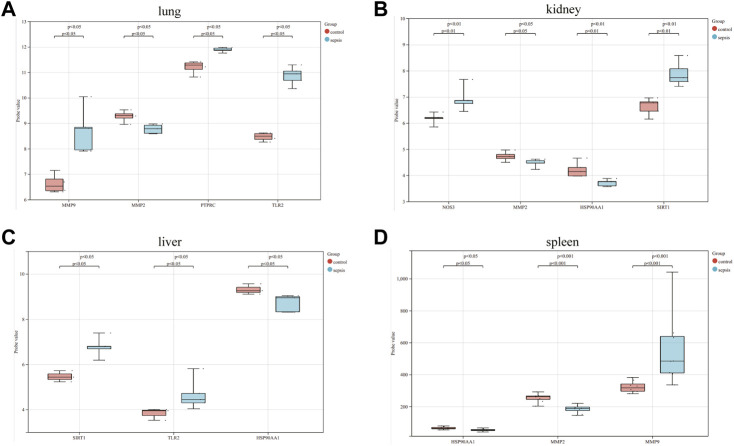
Eight hub genes in the sepsis liver injury-control group (SLi-C), sepsis lung injury-control group (SLu-C), sepsis kidney injury-control group (SKi-C), and sepsis Differential expression analysis in spleen injury-control group (SSp-C). Differential expression analysis of eight hub genes in the **(A)** sepsis lung injury-control group, **(B)** sepsis kidney injury-control group, **(C)** sepsis liver injury-control group, and **(D)** sepsis spleen injury-control group.

**FIGURE 10:**
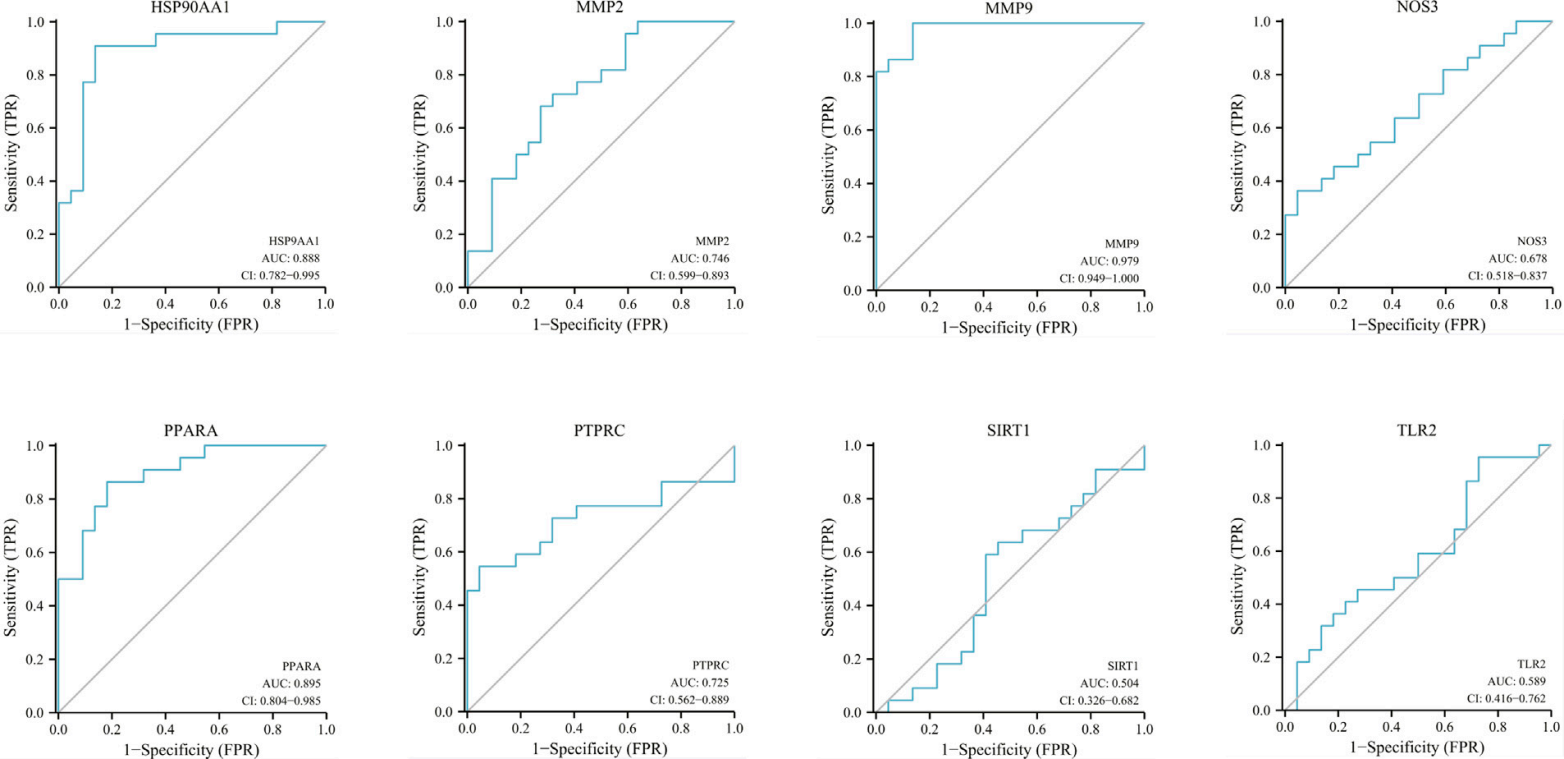
Diagnostic ROC curves for eight hub genes in differentiating sepsis from controls. ROC, receiver operating characteristic; AUC, area under the curve.

The correlation between hub genes and sepsis was analyzed by verifying the expression of eight hub genes in the dataset GSE95233 (Figure 8). Our results showed that MMP9, MMP2, PPARA, PTPRC, NOS3, TLR2, and HSP90AA1 were differentially analyzed in the sepsis survivor-control group (SS-C), sepsis non-survivor-control group (SN-C), *p*-value were all <0.05, there was the differential expression, indicating that key genes were related to the occurrence of sepsis. Among them, MMP9 was differentially expressed in the sepsis survivor-sepsis non-survivor group (SS-SN), indicating that MMP9 may be related to the prognosis of sepsis. There was no difference in the expression of SIRT1 between the SS-C and SN-C groups. The expression of key genes was verified in datasets GSE60088 and GSE5663, and the correlation between key genes and sepsis organ dysfunction was analyzed (Figure 9). Our results showed that MMP9, MMP2, PTPRC, and TLR2 were differentially expressed in the septic lung injury-control group (SLu-C), with a *p*-value < 0.05. Differential analysis of SIRT1, TLR2, and HSP90AA1 in the sepsis liver injury-control group (SLi-C), *p*-value were all <0.05, and differential expression. Differential analysis of NOS3, MMP2, HSP90AA1, and SIRT1 in the sepsis kidney injury-control group (SKi-C) showed that the *p*-value were all <0.05, indicating differential expression. Differential analysis of HSP90AA1, MMP2, and MMP9 in the sepsis spleen injury-control group (SSp-C) showed that the *p*-value were all <0.05, indicating differential expression. The analysis showed that NOS3, SIRT1, HSP90AA1, MMP9, MMP2, PTPRC, and TLR2 were associated with multiple organ damage in sepsis. The key genes were analyzed for sepsis diagnosis ability in the dataset GSE95233 (Figure 10). The AUCs of key genes in distinguishing sepsis from the control group were 0.888, 0.746, 0.979, 0.678, 0.895, 0.725, 0.504 and 0.589, respectively. The results showed that NOS3, SIRT1, and TLR2 had low diagnostic accuracy, HSP90AA1, MMP2, PTPRC, and PPARA had moderate diagnostic accuracy, and MMP9 had high diagnostic accuracy.

### 3.5 Molecular docking-autodock vina

**FIGURE 11:**
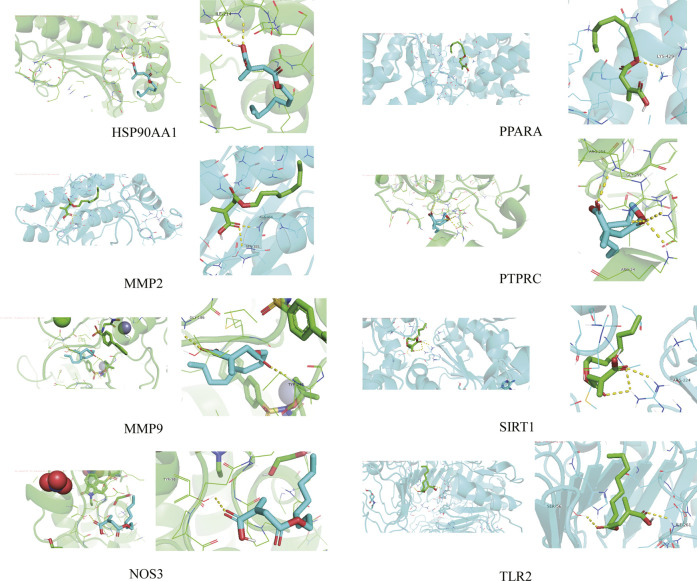
The visualization of 4-OI docking with eight Hub target molecules. The docking results of the Hub targets are displayed by PyMOL software, and the yellow dotted line represents the interaction line between the target and the compound.

The 4-OI was molecularly docked with the hub genes MMP9, MMP2, SIRT1, PPARA, PTPRC, NOS3, TLR2, and HSP90AA1, respectively. Each histone-ligand could spontaneously bind (binding energy <0 kcal/mol) according to the molecular docking binding energy. The binding energies of MMP9, MMP2, SIRT1, PPARA, PTPRC, and TLR2 were all ≤ -5 kcal/mol, indicating good protein-ligand binding. See Figure 11 for the visualization of molecular docking. The molecular docking details are shown in Table 3.

**TABLE 3:** 4-OI-Sepsis molecular docking.

Hub target	Compound	PDB ID	Binding Energy (KJ/mol-1)	Hydrogen bonding residues
TLR2	4-OI	2Z80	−5.4	SER-56, ILE-261
MMP9	4-OI	4H3X	−6.3	GLY-186, TYR-248
NOS3	4-OI	5D0C	−3.9	TYR-98
PTPRC	4-OI	4ZRT	−5.0	ARG-254, ARG-24, GLY-259
SIRT1	4-OI	4BVH	−5.5	ARG-224
HSP90AA1	4-OI	2YJW	−4.7	ILE-214
MMP2	4-OI	4WK7	−5.1	SER-355, ALA-356
PPARA	4-OI	2NPA	−6.3	LYS-429

### 3.6 Molecular docking-discovery studio

#### 3.6.1 Method reliability verification

Compared with the original crystal structures, the RSMD v1alues of MMP9, MMP2, SIRT1, PPARA, NOS3 and HSP90AA1 were 0.6750, 1.0058, 0.5717, 1.5777, 1.5056, 1.0859Å. The ligand conformation in the original crystal structure of the hub protein overlaps with the docked ligand conformation, and RMSD<2Å indicates that the calculation method can accurately predict the binding mode of the original ligand.

#### 3.6.2 Docking results

**FIGURE 12:**
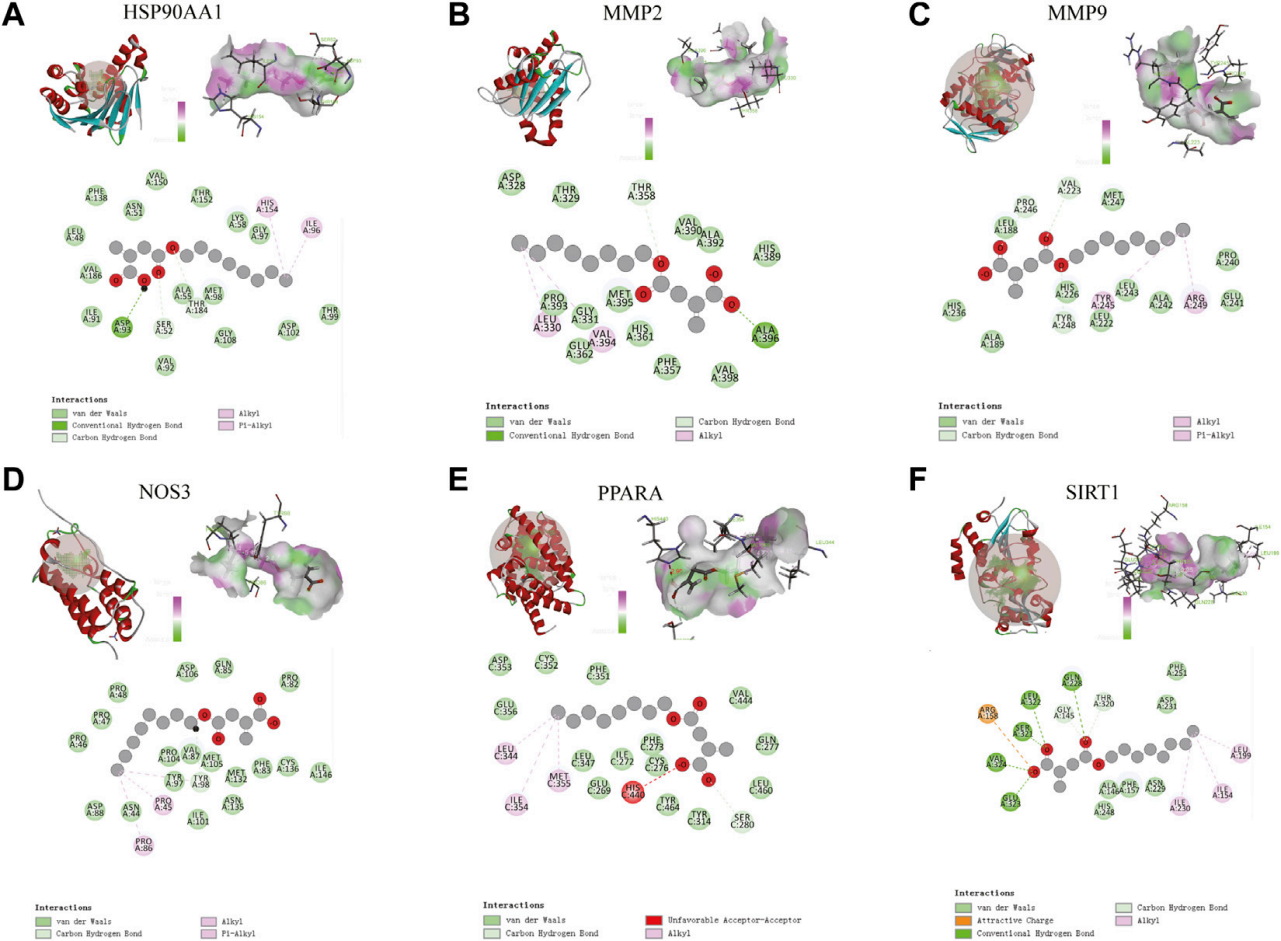
**(A–F)** Molecular docking results of MMP9, MMP2, SIRT1, PPARA, NOS3, and HSP90AA1 with 4-OI. (Upper left) 4-OI docking model with the hub target. (Top right) In the interaction diagram of 4-OI and the hub target hydrogen bond residues, the greener the color, the stronger the interaction force, and the more purple the color, the weaker the interaction force. (Bottom) Various interactions of 4-OI with hub targets, each color in the circle corresponds to a mode of action.

The molecular docking results of MMP9, MMP2, SIRT1, PPARA, NOS3, and HSP90AA1 with 4-OI are shown in Figure 12. AutoDock vina and Discovery Studio use different algorithms and scoring functions, which may cause differences in screening results. Combining two different algorithms, we believe that MMP9, MMP2, SIRT1, PPARA, NOS3, and HSP90AA1 are more likely to become hub targets. However, the molecular docking results of AutoDock vina software show that PTPRC and TLR2 bind well to small drug molecules. Therefore, we believe that PTPRC and TLR2 remain potential hub targets.

## 4 Discussion

Sepsis remains a global medical problem and the leading cause of death in intensive care units (ICU) inpatients. Microcirculation disturbance is one of the pathogenic mechanisms of sepsis and an important underlying cause of multiple organ failure (Pool et al., 2018). In the early stage of sepsis, impaired NO production reduces the number of perfused blood vessels, leading to microcirculation disturbance and aggravating sepsis (Wijnands et al., 2021). NOS3 is an endothelial nitric oxide synthase (eNOS) that protects vascular endothelial cells and maintains tight junctions of vascular endothelial cells. The dysfunction of NO production in the microcirculation during sepsis is mediated by NOS3 dysfunction (Chen et al., 2010). Decreased expression and activity of eNOS in cardiomyocytes can lead to myocardial dysfunction and death in sepsis (Ichinose et al., 2007). In addition, an animal study showed that the over-activation of eNOS expression could reduce the mortality of animals with LPS-induced sepsis (Hollenberg and Singer, 2021). Peroxisome proliferator-activated receptor-α(PPARα) is a nuclear receptor protein that regulates transcription factor expression, which can be involved in many biological processes, including energy metabolism, cell growth, and apoptosis. The study has shown that targeting PPARα can improve septic kidney injury and the survival rate of septic rats (Wang et al., 2020). It has been reported that increasing the expression of PPARα can protect HK2 and HEK293 cells from LPS-induced damage and inhibit apoptosis and oxidative stress (Hu et al., 2021). Meng et al. (2011) showed that the activation of AMPK, an important upstream signaling molecule of PPARα, can activate PPARα to enter the nucleus and regulate the transcription of related inflammation-related factors, such as TNF-α and IL-1β. Matrix metalloproteinases (MMPs) regulate inflammatory responses by remodeling blood vessels and degrading the extracellular matrix. Among them, MMP2 and MMP9 are important genes involved in the inhibition and activation of platelet production, and thrombocytopenia is an adverse indicator associated with a significant increase in the risk of death in the development of sepsis (Larkin et al., 2018). Studies have shown that plasma levels of MMP-2 and MMP-9 are elevated in patients with sepsis. The increased release of MMP-2 and MMP-9 promotes the formation of platelet-leukocyte aggregates (PLAs) and the formation of PLAs It contributes to the formation of microthrombi and the occurrence of microvascular disorders in sepsis, aggravating sepsis (Kirschenbaum et al., 2000; Chung et al., 2004; Larkin et al., 2018). Knockout of the MMP9 gene in a mouse model of sepsis can effectively reduce the inflammatory response and improve the survival rate of mice with sepsis which may be related to inhibiting the secretion of inflammatory cytokines (Chen et al., 2020). One study showed that downregulating the expression of MMP-2 and MMP-9 by regulating the ERK1/2 signaling pathway alleviated LPS-induced cardiac injury and dysfunction (Han et al., 2017). Protein tyrosine phosphatase receptor C(PTPRC) is a receptor-type phosphatase abundantly expressed on all nucleated hematopoietic cells that regulate diverse signaling pathways, including signaling, neutrophil recruitment, and ROS production (Zhu et al., 2011). German et al. found that PTPRC plays a key role in leukocyte recruitment and bacterial clearance during *Escherichia coli* lung infection (Germena et al., 2015). HSP90AA1 is a member of the heat shock protein 90α family, which regulates biological processes such as cellular oxidative stress and signal transduction. HSP90α is thought to play an important role in wound repair, inflammatory response, and stress. HSP90α protein can induce inflammation by activating the NF-kB pathway and STAT3 transcriptional program and promote the production of inflammatory cytokines (including IL-6 and IL-8); at the same time, activated NF-kB can induce the expression of HSP90α, leading to the production of inflammatory factor storm (Bohonowych et al., 2014). TLR2 is a pattern recognition receptor with a broad recognition spectrum, which can recognize most pathogens, microorganisms, and bacterial endotoxins, and participate in inflammatory activation and immune response. Knockdown or inhibition of TLR2 expression has been reported to attenuate mitochondrial dysfunction and reduce chemokine production in LPS-induced sepsis, reducing organ dysfunction in sepsis (Zuo et al., 2020). In addition, NF-κB and AP-1 transcription factors can be activated by targeting the TLR2/MyD88 axis to initiate inflammatory response-related genes, such as TNF-α, IL-1β, and IL-6 (Castoldi et al., 2012). SIRT1 is an NAD+-dependent protein deacetylase discovered in recent years. When the body is in a state of systemic inflammatory response, the SIRT1 gene is activated and inhibits the release of inflammatory factors by regulating various inflammatory signaling pathways (Li et al., 2019). It plays an important role in apoptosis, anti-oxidative stress, anti-inflammatory, and anti-tumor. It was found that inhibiting apoptosis and increasing autophagy by activating SIRT1 expression protected cardiac dysfunction in septic mice (Zhang et al., 2019).

The anti-sepsis pathway of the core, as mentioned above genes, is mainly focused on the regulation of immune and inflammatory responses, which is also in line with the pathophysiological development of sepsis. The over-activation of the inflammatory response in the early stage of sepsis to achieve the purpose of clearing pathogens and the late development of an immunosuppressive state to promote tissue repair, once the imbalance between the excessive inflammation and the immunosuppressive state occurs, it will lead to sepsis patients organ dysfunction and death (Fitzpatrick, 2019). Sepsis is a life-threatening organ dysfunction caused by the dysregulation of systemic inflammatory response. However, the pathogenesis of sepsis cannot be entirely attributed to an abnormal inflammatory response. It may also be related to complex disorders of immune metabolism, which is one of the reasons that hinder the development of effective drugs for sepsis (Weis et al., 2017; Koutroulis et al., 2019). In addition, disease tolerance is becoming a hub strategy for treating sepsis, and its mechanisms involve aspects such as immune metabolism, immune fitness, inflammation, and genetics (Appiah et al., 2021). Relevant studies have shown that the innate and adaptive immune systems play an essential role in disease tolerance defense (McCarville and Ayres, 2018). Itaconate is one of the most highly induced metabolites in activated macrophages, which regulates the production of early cytokines and participates in the establishment of disease tolerance. It regulates succinate levels and functions, mitochondrial respiration, and ROS levels.

Moreover, the production of inflammatory cytokines regulates inflammatory and immunometabolic responses (Lampropoulou et al., 2016; Domínguez-Andrés et al., 2019). Considering the low cell permeability of itaconate, further studies on the biological function of itaconate cannot be satisfied, which prompted the development of itaconate derivatives with cell permeability, such as 4-octyl itaconate (4-OI), Dimethyl itaconate (DI) and Ethyl itaconate (4-EI), the relevant information of itaconate and its derivatives is shown in Table 4. 4-OI is a highly electrophilic, cellular Permeability and alkylation of cysteine residue derivatives on various proteins make it a better candidate for the study of itaconate (Henderson et al., 2021). As DI with the same high electrophilicity and cell permeability as 4-OI. Zhang et al. (2021) found that DI could reduce the inflammatory response and sepsis by regulating the expression of the Nrf2 signaling pathway in LPS-induced macrophages. In addition, DI can treat IL-17-IκBζ-mediated autoimmune diseases such as psoriasis (Bambouskova et al., 2018). However, compared with 4-OI, since DI only increases itaconate synthesis and cannot be converted into endogenous itaconate, this may be related to its more straightforward hydrolysis by esterases (Lin et al., 2021). In conclusion, we predict that 4-OI may protect against sepsis by regulating the balance of inflammatory and immune-related signaling pathways. However, 4-OI is also not a perfect substitute for itaconate derivatives. In the study of Swain et al. (2020), itaconate derivatives may not lead to the expected accumulation of itaconate in cells, leading researchers to misinterpret the derivative-related data. Furthermore, there are some differences in electrophilic and immunological properties between the native form of itaconate and its derivative (4-OI), which may not recapitulate the role of endogenous itaconate well. Therefore, we should also further search for more suitable derivatives in the future.

**TABLE 4:** The information sheet of itaconate and its derivatives .

	Itaconate	4-Octyl itaconate (4-OI)	Ethyl itaconate (4-EI)	Dimethyl itaconate (DI)
Molecular formula	C_5_H_6_O_4_	C_13_H_22_O_4_	C_7_H_10_O_4_	C_7_H_10_O_4_
SMILES	C = C [CC( = O)O]C ( = O)O	CCCCCCCCOC( = O)CC( = C)C ( = O)O	CCOC( = O)CC( = C)C (=O)O	COC(=O)CC(=C)C ( = O)OC
Molecular weight	130.10	242.31	158.15	158.15
Electrophilicity	±	++	±	++
PubChem CID	811	14239884	533740	69240
Hydrogen bond donor count	2	1	1	0
Hydrogen bond acceptor count	4	4	4	4

In the GO and KEGG analysis of hub genes, the anti-septic effect of 4-OI may be related to the regulation of Macrophage differentiation, Nitric oxide synthase activity, and Neutrophil degranulation, Protein phosphorylation, response to hypoxia, IL-17 signaling pathway, PI3K-Akt signaling pathway. 4-OI is an essential regulator of metabolic reorganization in inflammatory macrophages. In a sepsis model, 4-OI can improve the survival rate of septic mice by inhibiting GAPDH activity and reducing glycolysis, inhibiting the activation of inflammatory macrophages and the release of inflammatory cytokines (Liao et al., 2019). Li et al. found that the activation of the IL-17 signaling pathway can promote the occurrence of sepsis (Li et al., 2020). In the *in vitro* and *in vivo* model of sepsis, the up and down molecules of the IL-17 signaling pathway (HMGB1, RAGE, IL-17A, NK-κB) and inflammatory factors (IL-1β, IL-18) were upregulated. There are some studies have shown that activation of the PI3K/Akt pathway can promote the expression of heme oxygenase-1 (HO-1), which in turn plays an anti-apoptotic or anti-inflammatory role in the oxidative damage response and improves multiple organ dysfunction in sepsis (Kim et al., 2018; Xiao et al., 2018). In addition, 4-OI can regulate inflammation through multiple pathways, including the KEAP1-Nrf2 pathway, Nf-kb/ATF3 pathway, and toll-like receptor pathway. Using this robust network pharmacology and bioinformatics approach (Hopkins, 2007; Chen et al., 2020), our study found that the anti-septic effect of 4-OI may be closely related to regulating inflammatory response, immune system, oxidative stress, and various metabolisms. It exerts its anti-septic effect through multiple pathways. In addition, our analysis results show that 4-OI has a particular application value in the treatment of prostate cancer, bladder cancer, atherosclerosis, and endocrine diseases. Therefore, 4-OI may be a potentially valuable drug.

Considering that sepsis is still a significant problem facing the global medical system, we used network pharmacology to explore the hub genes and mechanisms of 4-OI anti-sepsis for the first time. We then verified their feasibility and possibility through molecular docking technology. The docking site will provide a reference for studying the molecular mechanism of 4-OI in treating sepsis. It will significantly reduce the time and cost of new drug applications. Although studies have reported the molecular mechanism of 4-OI in treating sepsis, the molecular mechanism of 4-OI anti-sepsis exploration through network pharmacology has not yet been reported.

## 5 Conclusion

In conclusion, our study identified central genes associated with 4-OI anti-sepsis and validated the reliability of predicted central targets using molecular docking techniques. The enrichment analysis results indicated that immune dysfunction and abnormal activation of inflammatory response were the potential action pathways of 4-OI against sepsis. However, our study also has certain limitations. First, in future work, the Hub gene and its pharmacological mechanism of 4-OI in the treatment of sepsis still need to be verified by *in vitro* and *in vivo* models, which will be the focus of our future research. Second, whether the pharmacological effects of itaconate are as helpful in human patients as in animal and experimental cellular models is also an issue that we still need to address. We will further validate the feasibility of predicting key targets in clinical trials in follow-up studies. This study provides new supplements and ideas for the molecular mechanism of 4-OI in the treatment of sepsis.

## Data Availability

The original contributions presented in the study are included in the article/Supplementary Material, further inquiries can be directed to the corresponding authors.

## Abbreviations

4-OI, 4-octyl itaconate; DEGs, differentially expressed genes; GAPDH, glyceraldehyde - 3 - phosphate dehydrogenase; GEO, gene expression omnibus; GO, gene ontology; KEGG, kyoto encyclopedia of genes and genomes; KOBAS, KEGG orthology based annotation system; LPS, lipopolysaccharide; PDB, protein data bank; PPI, protein-protein interaction; SDF, spatial data file; SEA, similarity ensemble approach; STP, swiss targetprediction.
